# Flow Velocity Measurement Using a Spatial Averaging Method with Two-Dimensional Flexural Ultrasonic Array Technology

**DOI:** 10.3390/s19214786

**Published:** 2019-11-04

**Authors:** Lei Kang, Andrew Feeney, Riliang Su, David Lines, Sivaram Nishal Ramadas, George Rowlands, Steve Dixon

**Affiliations:** 1 Department of Physics, University of Warwick, Coventry CV4 7AL, UK; l.kang.1@warwick.ac.uk (L.K.); a.feeney@warwick.ac.uk (A.F.); g.rowlands@warwick.ac.uk (G.R.); 2 Department of Electronic and Electrical Engineering, University of Strathclyde, Glasgow G1 1XW, UK; suriliang2004@163.com (R.S.); david.lines@strath.ac.uk (D.L.); 3 Honeywell, Reddidtch B98 9ND, UK; nishal.ramadas@honeywell.com

**Keywords:** transit-time ultrasonic flow measurement, flow velocity, spatial averaging, flexural ultrasonic array transducer

## Abstract

Accurate average flow velocity determination is essential for flow measurement in many industries, including automotive, chemical, and oil and gas. The ultrasonic transit-time method is common for average flow velocity measurement, but current limitations restrict measurement accuracy, including fluid dynamic effects from unavoidable phenomena such as turbulence, swirls or vortices, and systematic flow meter errors in calibration or configuration. A new spatial averaging method is proposed, based on flexural ultrasonic array transducer technology, to improve measurement accuracy and reduce the uncertainty of the measurement results. A novel two-dimensional flexural ultrasonic array transducer is developed to validate this measurement method, comprising eight individual elements, each forming distinct paths to a single ultrasonic transducer. These paths are distributed in two chordal planes, symmetric and adjacent to a diametral plane. It is demonstrated that the root-mean-square deviation of the average flow velocity, computed using the spatial averaging method with the array transducer is 2.94%, which is lower compared to that of the individual paths ranging from 3.65% to 8.87% with an average of 6.90%. This is advantageous for improving the accuracy and reducing the uncertainty of classical single-path ultrasonic flow meters, and also for conventional multi-path ultrasonic flow meters through the measurement via each flow plane with reduced uncertainty. This research will drive new developments in ultrasonic flow measurement in a wide range of industrial applications.

## 1. Introduction

The measurement of average velocity of flowing fluids is vital for flow measurement, which has become increasingly essential for many applications in the oil and gas, aerospace, automotive, power, chemical, and pharmaceutical process industries, in addition to those for a range of military and government operations [1,2,3,4]. Accurate and versatile flow measurement is essential to these industries to ensure fluid characteristics are known in complex flow systems, for example, in the interests of safety, enabling the condition of the fluid within a system to be continuously monitored, to improve the effectiveness or efficiency of fluid flow systems, including the management of energy demand, and to understand the influence of environmental parameters such as pressure or temperature on a fluid flow system. Flow measurement can be a complex process where a compromise is necessary between the quality of measurement data and the disruptive influence of a flow measurement system within the flow medium to be monitored. The properties of flowing fluids can vary significantly from one medium to another. Therefore, a range of flow meters have been developed, operating according to various physical principles and different levels of complexity [1,2,3,4,5,6,7,8,9,10,11,12,13,14,15,16,17,18,19,20,21,22,23,24,25,26,27]. Among them, the ultrasonic transit-time flow measurement method has been successfully applied in industrial and fiscal metering for many years with numerous key advantages. For example, it is principally non-intrusive, there are no moving parts required, it exhibits a high turndown ratio (measurable range of flow), and incorporates a bidirectional flow measurement capability [2].

To ensure the accuracy of measurement, various configurations of ultrasonic paths, defined by the positions of ultrasonic transducers working in a pitch–catch mode, have been devised and commercialized for single-path and multi-path transit-time ultrasonic flow meters [1,2,3,4,8,9,10,11,12,13,14,15,16,17,18,19,20,21,22,23]. For an ideal flow velocity profile without any disturbance, its velocity vectors are axisymmetric about the axis of a pipe. The average velocity of the flowing fluids can be deduced based on an ultrasonic transit-time measurement via a single ultrasonic path in a diametral plane with a reasonable accuracy [9]. Multiple ultrasonic paths situated respectively in different diametral and chordal planes have also been utilized to extract the velocity information from different sections of the velocity profile [11,12,13,14,15,16,17,18,19,20,21,22,23]. By adopting appropriate weighted integration algorithms and by assigning accurate weightings to the results measured via different paths, the overall average flow velocity can thereby be deduced. It is also possible with multi-path configurations to arrange one or more pairs of ultrasonic paths in a symmetric manner. This means that the circumferential flow, for example, the effect of swirl on the ultrasonic transit-time measurement, can be significantly suppressed by the summation of the individual measurements obtained via the symmetric planes, leading to a further improvement of measurement accuracy [13,14]. As the average velocity measured by a multi-path flow meter is a combination of several measured velocities respectively extracted via multiple ultrasonic paths from different diametral and/or chordal planes, its accuracy is generally higher than that measured by a single ultrasonic path flow meter. However, the overall accuracy from both single-path and multi-path transit-time ultrasonic flow meters relies principally on the velocity information measured via each ultrasonic path, as well as the actual velocity profile of the flowing fluids. Another approach that has been proposed to obtain a representative profile of flow velocity is based on a computed tomography technique [24,25], but the accuracy of the reconstructed profile is also highly dependent on the reconstruction algorithm, the number of ultrasonic paths, the spatial distribution of the paths, and the accuracy of each flow velocity measurement from the individual ultrasonic paths.

Although the transit-time ultrasonic flow meters in the configurations discussed above have been suited to a range of industrial and fiscal applications and requirements for some time, there remains a strong demand for the improvement of the measurement accuracy of flow meter systems. In practice, flowing fluids may contain swirls, pulsations, eddy currents, vortices, or turbulence, and the velocity profile can be asymmetric. There are numerous possible sources of error, which can affect flow velocity measurement and increase measurement uncertainty [18]. One common source is from time delays associated with the measurement system, for example, through delays in transmitting and receiving transducers, or from cabling or electronic circuitry. To a certain extent, these delays can generally be compensated for in the calibration process [18], but the compensation is most effective for relatively steady, non-turbulent, and non-disturbed flow. Another source of error can be attributed to dynamic phenomena, such as the dynamic fluctuations of ultrasonic waves due to fluid dynamic effects via stochastic swirls, vortices, eddy currents, or turbulence in the flow, all of which have been investigated through numerical simulation and experiments [18,26,27]. Since most conventional single-path and multi-path ultrasonic flow meters tend to extract the velocity information of each flow plane (either diametral or chordal) via only a single or two symmetric ultrasonic paths, the dynamic fluctuations of ultrasonic waves can cause temporal errors in the measurement of average flow velocity for each individual ultrasonic path. This leads to a degradation in the accuracy and quality of the overall flow velocity measurement. Consequently, there is evidently a significant requirement for novel strategies to enhance the accuracy of flow velocity measurement.

Recently, novel ultrasonic flow measurement systems based on flexural ultrasonic phased array transducers were devised and reported [28,29,30,31]. The beam-steering technique has been applied either to the transmitting process or to the receiving process of the phased array transducer, where air-coupled flexural ultrasonic phased array technology was shown to be inexpensive and robust, with low-voltage operation and each array element being able to generate a relatively broad ultrasound radiation pattern [28,29,30,31]. In this research, a two-dimensional flexural ultrasonic array transducer, whose structure is similar to that of the phased array transducer, but where each array element operates individually and independently, is further investigated. A mathematical method of spatial averaging is used, which takes full advantage of the flow measurement data obtained by the two-dimensional array transducer. We show that individual ultrasonic signal measurements in a single flow measurement process can be coupled together, in order to reduce the influence of dynamic fluctuations and swirls in fluid flow and enhance the accuracy of flow velocity measurement inside a fluid channel, for a more reliable measurement of flow. The combination of the spatial averaging method with two-dimensional flexural ultrasonic array technology will be beneficial for improving the accuracy of ultrasonic flow measurement in different classes of single-path and multi-path ultrasonic flow meters and for a wide range of industrial environments and applications.

## 2. Methodology

**Figure 1:**
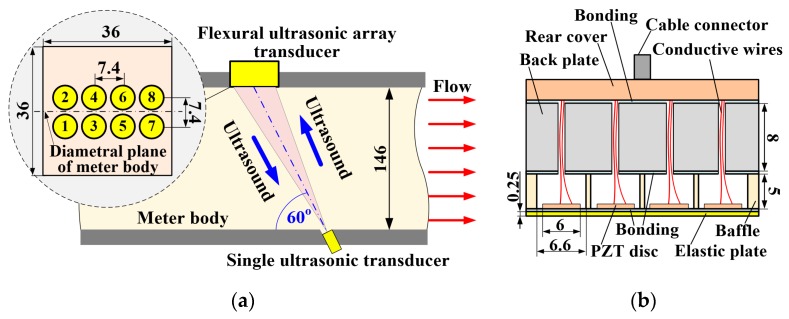
Schematic diagrams of the flow meter system, showing (**a**) the position of the ultrasonic transducers inside the meter body, and (**b**) the construction of the flexural ultrasonic array transducer. All dimensions are in millimeters.

A flow meter body with a diameter of 146 mm was designed and fabricated to accommodate a two-dimensional flexural ultrasonic array transducer and a commercial single ultrasonic transducer (PROWAVE 500MB120, Pro-Wave Electronic Corporation, New Taipei City, Taiwan) [28]. The assembly schematic and associated dimensions of the flow meter system are shown in Figure 1a, where the flexural ultrasonic array transducer comprises eight individual flexural ultrasonic elements in a 2 × 4 configuration, in which the two rows of elements are positioned along the axial direction of the meter body, symmetric and adjacent to the diametral plane.

The single transducer is fixed in the same diametral plane as the flexural ultrasonic array transducer, where its centre faces that of the array at an angle of 60° from the wall of the meter body. The array transducer consists of an elastic titanium plate, a stainless steel baffle, a stainless steel backplate, and eight piezoelectric ceramic discs, all bonded using a two-component epoxy (Araldite 2014), as illustrated in Figure 1b. The dimensions of the elastic plate are 36 × 36 × 0.25 mm, and the dimensions of the baffle are 36 × 36 × 5 mm. Eight apertures, each with a diameter of 6.6 mm and a pitch of 7.4 mm, are machined into the baffle, thereby defining the boundary condition of the flexural vibration of each array element. The dimensions of the backplate are 36 × 36 × 8 mm. The backplate creates an enclosed cavity for each array element, further strengthening the rigid boundary condition of array elements and enhancing the mechanical and electrical robustness of the array transducer [28]. The eight array elements are sequentially numbered from one to eight, where each element can operate independently with the single transducer, thereby representative of a classical pitch–catch configuration. Therefore, this system comprises eight individual ultrasonic paths.

**Figure 2:**
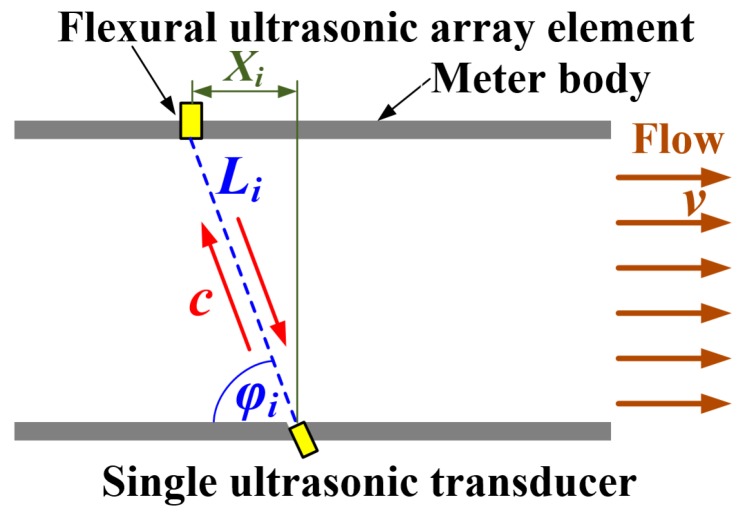
The principle of the transit-time ultrasonic flow measurement method.

For reference, the transit-time ultrasonic flow measurement method is schematically illustrated in Figure 2 in the context of this research, where only one array element and its corresponding ultrasonic path are shown for simplicity. In Figure 2, c represents the speed of ultrasound in the fluid, v denotes the average flow velocity of the fluid over the cross-sectional area of the meter body, $X_{i}$ stands for the axial distance between the centres of the *i*-th array element and the single transducer, $\varphi_{i}$ denotes the angle of the ultrasonic propagation path to the wall of the meter body, and $L_{i}$ represents the true distance between the centre of the single transducer and the *i-*th array element. A simultaneous equation can be formed by Equation (1) to describe the relationship between the propagation distance of ultrasound $L_{i}$, the speed of ultrasound c, average flow velocity along the ultrasonic path $v_{L,i}$, and the time of flight (ToF) of ultrasound travelling downstream $t_{d,i}$ and upstream $t_{u,i}$ [1,2,3,4].

(1) $$\{\begin{array}{c} L_{i}=\left(c+v_{l,i}\times \cos\varphi_{i}\right)\times t_{d,i}\;\\ L_{i}=\left(c-v_{l,i}\times \cos\varphi_{i}\right)\times t_{u,i} \end{array}$$

Then, $v_{L,i}$ can be determined by elimination of the ultrasonic velocity c in Equation (1), as shown in Equation (2).

(2) $$v_{L,i}=\frac{L_{i}^{2}\left(t_{u,i}-t_{d,i}\right)\;}{2X_{i}t_{u,i}t_{d,i}}$$

In practice, the distribution of flow velocity of fluids in a pipe cannot be considered uniform. For a steady, fully developed velocity flow free from swirl and pulsation, the velocity profile is symmetric about the axis of the pipe, and the velocity at a given point inside the pipe is a function of the distance between the location of that point and the axis of the pipe [1,2,3,4]. Consequently, a correction factor $K_{c,i}$ is introduced to estimate the average velocity $v_{A,i}$ over the cross-sectional area based on the measured average velocity $v_{l,i}$ along the ultrasonic path, as shown in Equations (3) and (4) [18].

(3) $$K_{c,i}=\frac{\frac{1}{A}\iint_{A}^{}v\left(r\right)\;dA}{\frac{1}{L_{i}}\int_{L_{i}}^{}v\left(r\right)\;dL_{i}}$$

(4) $$v_{A,i}=K_{c,i}v_{L,i}$$

The cross-sectional area of pipe A, the velocity profile v(r), which is a function of the Reynolds number, and the distance from the axis of pipe r are all vital parameters for this calculation. Consequently, the average flow velocity $v_{A}$ over the cross-sectional area measured by the eight ultrasonic paths can be expressed by Equation (5).

(5) $$v_{A}=\frac{1}{8}\overset{8}{\underset{i=1}{\sum }}K_{c,i}v_{L,i}$$

**Figure 3:**
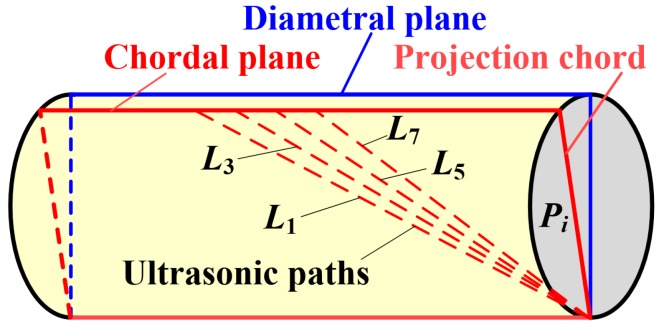
Illustration of the individual ultrasonic paths and their corresponding projection chords on the cross-sectional area of a pipe, showing that ultrasonic paths (1,3,5,7) are in the same chordal plane and correspond to the same correction factor in a fully developed, undisturbed flow profile.

In a fully developed and undisturbed flow profile without swirl or pulsation, the flow velocity profile at any cross-section of a pipe is constant, and therefore, the complete average flow velocity determined from the velocities of the individual ultrasonic paths is equivalent to the average flow velocity along their corresponding projection chords on the cross-sectional area of the pipe, as illustrated in Figure 3. Only the four ultrasonic paths defined by the array elements (1,3,5,7) are shown in Figure 3 for clarity. Therefore, Equation (3) can also be expressed as Equation (6), where $P_{i}$ is the projection chord of the ultrasonic path $L_{i}$ on the cross-sectional area of the pipe.

(6) $$K_{c,i}=\frac{\frac{1}{A}\iint_{A}^{}v\left(r\right)\;dA}{\frac{1}{P_{i}}\int_{P_{i}}^{}v\left(r\right)\;dP_{i}}$$

As the ultrasonic paths (1,3,5,7) between the single transducer and the array elements (1,3,5,7) have the same projection chord, their associated correction factors are identical. Likewise, paths (2,4,6,8) also require identical correction factors. Furthermore, the ultrasonic paths (1,3,5,7) and paths (2,4,6,8) are symmetric about the diametral plane of the pipe. Therefore, in a fully developed undisturbed flow without swirl or pulsation, the eight ultrasonic paths between the single ultrasonic transducer and the eight array elements have identical correction factors due to the axisymmetric nature of the velocity profile. Assuming the correction factor is $K_{c}$, then Equation (5) can be further simplified as Equation (7).

(7) $$v_{A}=\frac{K_{c}}{8}\overset{8}{\underset{i=1}{\sum }}v_{L,i}$$

In essence, Equation (7) is a three-dimensional spatial averaging of flow velocity, determined via velocity measurements from the eight ultrasonic paths. Using this relationship, the axial flow velocity of the medium flowing through the three-dimensional section constituting the eight paths is spatially averaged, whilst the fluctuation in the eight measurements due to the stochastic disturbance of vortices, eddy currents, and turbulence along these individual paths can be suppressed [32]. In addition, because the eight ultrasonic paths are symmetric about the diametral plane, the influence of circumferential flow (swirls) can also be suppressed [2,13,14]. The principles underlying the approach defined in this section can thus be employed in the processing of experimental flow measurement data.

## 3. Implementation of the Spatial Averaging Strategy

**Figure 4:**
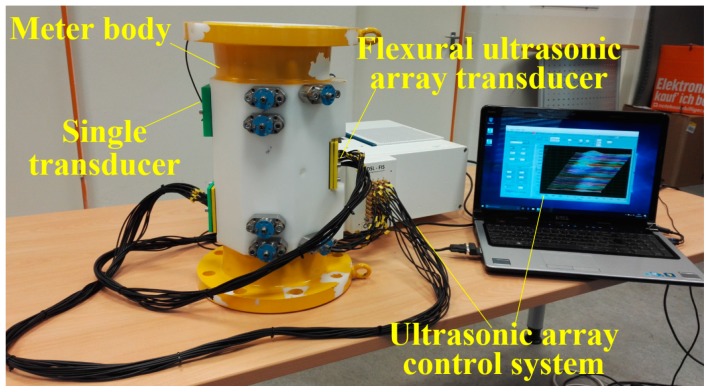
The prototype for the ultrasonic flow meter, where the control system digitizes, records, and processes the ultrasonic signals.

A 32-channel ultrasonic array control system (FIToolbox, Diagnostic Sonar, Livingston, UK) controls the generation and detection of the ultrasonic transducers, enabling the acquisition of ultrasonic signals at different flow rates. The control system, which comprises the single transducer and flexural ultrasonic array transducer, is shown alongside the meter body in Figure 4 [29].

**Figure 5:**
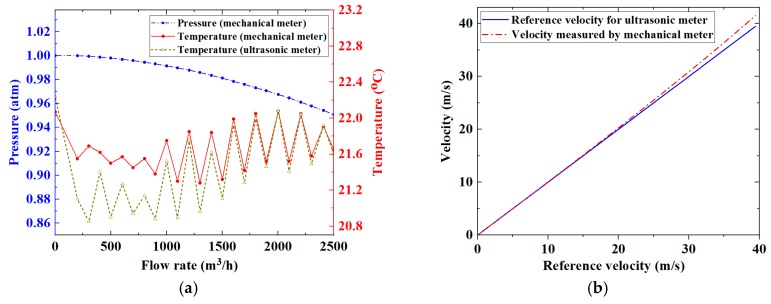
(**a**) Variations of pressure and temperature in the calibrated reference mechanical meter and the ultrasonic flow meter for different flow rates, where the ultrasonic meter pressure is a constant 1 atm; and (**b**) the comparison between flow velocity measured by the reference mechanical meter and the deduced reference flow velocity for the ultrasonic flow meter.

Flow experiments were conducted with a commercial flow rig (Honeywell Process Solutions, Mainz, Germany). The rig consisted of a compressor (HVM 80-125 GR, Venti Oelde, Oelde Germany) as a flow source and a calibrated reference mechanical flow meter (TRZ G1600 DN200, Elster Instromet, Mainz, Germany) as a reference meter. All experiments were conducted in an open flow loop using air as the flowing medium. A 49 kHz, 5-cycle sine wave tone burst signal with an amplitude of 20 V_P-P_ was chosen as the driving signal. The eight array elements and the single transducer sequentially transmitted and received ultrasonic waves with a repetition frequency of 100 Hz. To enhance the signal-to-noise ratio of the received ultrasonic signals, 8-time averaging was applied to each measurement. The ultrasonic signals travelling through the eight paths upstream and downstream were digitized and recorded by the FIToolbox at different flow rates, ranging from 0 to 2500 m^3^/h in increments of 100 m^3^/h, measured by the reference mechanical flow meter. Temperatures and pressures in the ultrasonic and the reference mechanical flow meters at different flow rates were recorded by the commercial flow rig, the results for which are shown in Figure 5a, where the pressure in the vicinity of the ultrasonic flow meter is a constant 1 atm and is not shown. The reference flow rates and the reference average flow velocity over the cross-sectional area of the meter body were determined according to the ideal gas law. These results are shown in Figure 5b.

The speed of ultrasound *c* in air is approximately a function of temperature *T* in degrees Celsius (°C), as shown in Equation (8) [33].

(8) $$c\approx 331.45\sqrt{1+\frac{T}{273}}\;(m/s)$$

**Figure 6:**
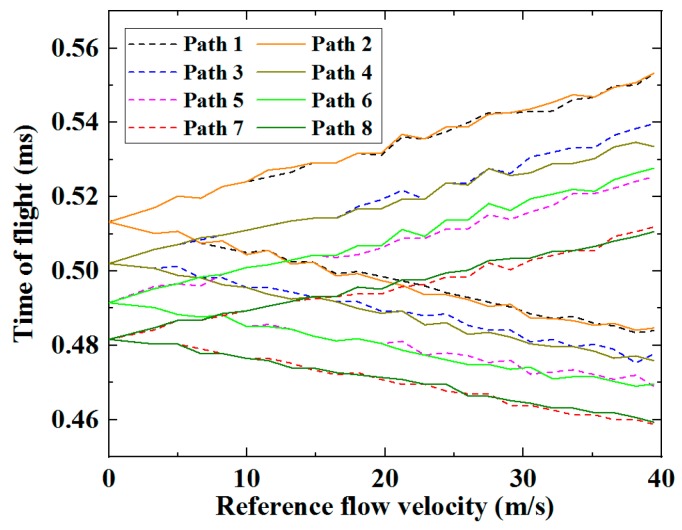
ToFs for ultrasonic waves travelling through the eight paths upstream and downstream at different flow velocities.

The ultrasonic speed was estimated according to Equation (8) for the zero-flow condition; and thus, the ToF for each ultrasonic path could be calculated based on the length of its respective corresponding path. The differences in ToF between non-zero and zero flow conditions for each ultrasonic path were respectively calculated by the FIToolbox system based on cross-correlation. Therefore, the ToF of each path at different flow rates could be determined [9], and these are shown in Figure 6.

**Figure 7:**
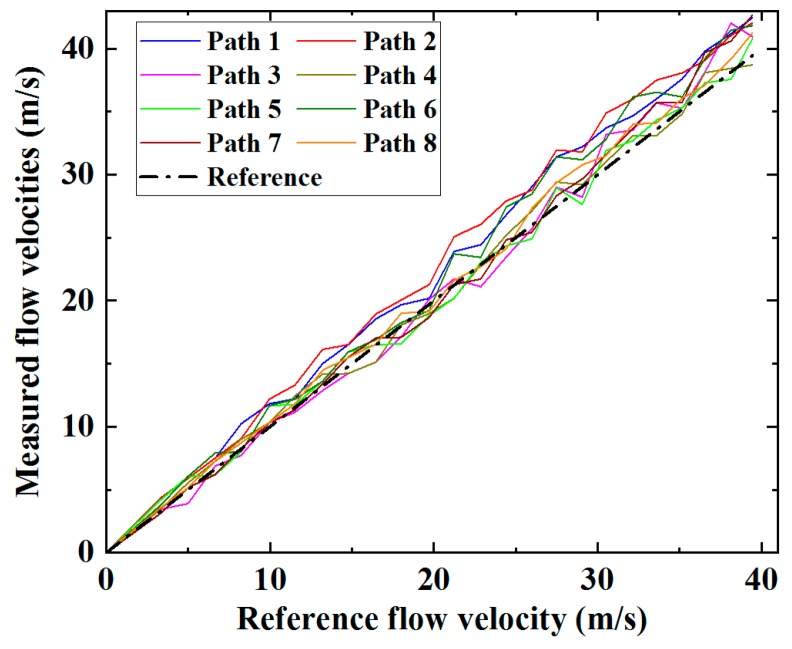
Average flow velocities along the eight paths determined through the ultrasonic transit-time method, showing a strong correlation between measurement and reference results.

As expected, the ToFs increased when the ultrasound waves travel upstream and decreased when travelling downstream. Fluctuations in the magnitude of ToF were observed in all paths, for both downstream and upstream cases. In an undisturbed fully developed flow without swirls or pulsation, the ToF of the ultrasonic waves travelling through ultrasonic paths with the same length, for example Paths 1 and 2, should be identical. The fluctuations shown in Figure 6 are a strong indicator of disturbance in the flow velocity profile caused by vortices, eddy currents, swirls, and turbulence of the flow [18,26,27]. Using Equation (2), the average flow velocities along the eight paths were respectively calculated based on their corresponding ToFs, individually measured via the eight independent ultrasonic paths, and are shown in Figure 7.

The measured velocity increased with the reference flow velocity, consistent for all eight paths. However, a growing discrepancy is observable and as expected, the measured flow velocities show a general tendency to be higher compared to the reference velocities. This is principally due to the discrepancy between the average flow velocity determined from measurements of the individual ultrasonic paths and the average velocity over the cross-section of the meter body [2]. Fluctuations in the data trends are observed for all paths measured at all flow rates, and a key consequence of this is that it increases the root-mean-square (RMS) deviation of the measurement results from the reference flow velocity. Since all array elements are in the vicinity of the diametral plane, the correction factor can be estimated using the correction factor for the paths travelling through the diametral plane. For a fully developed turbulent flow, the correction factor for the ultrasonic paths in the diametral plane is a function of the Reynolds number, and can be estimated by (9) [18].

(9) $$K_{c}\approx \frac{1}{1.12-0.011\log_{10}Re}$$

**Figure 8:**
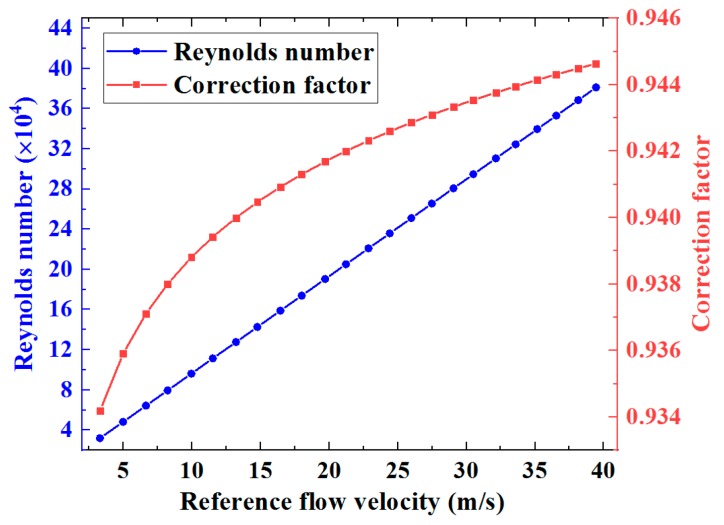
Reynolds number and correction factor as functions of reference flow velocity.

**Figure 9:**
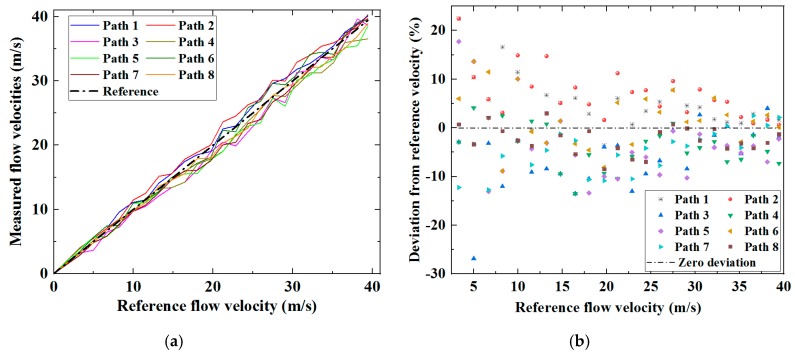
(**a**) Average flow velocity measured by the ultrasonic flow meter and (**b**) the deviation of this measurement from the reference.

Using Equation (9), the Reynolds number and the correction factor are both shown in Figure 8 as functions of the reference flow velocity, and the average flow velocities over the cross-section of the ultrasonic flow meter body can be estimated from the average flow velocities along the individual ultrasonic signal paths via Equation (4), as shown in Figure 9. The measured average cross-sectional flow velocities from the eight ultrasonic paths correlate closely with the reference velocities. The improvement can be compared with the results shown in Figure 7. There remain fluctuations associated with fluid dynamic effects in the results shown in Figure 9a, but these are unavoidable physical phenomena. For a fully developed flow profile without any swirl or other types of disturbance, the average flow velocity respectively measured through the eight ultrasonic paths in two chordal planes symmetric about the diametral planes should be identical. The differences between flow velocities measured via eight ultrasonic paths exhibited in Figure 9a imply that measuring flow velocity via only a single ultrasonic path in each plane can increase uncertainty in flow measurement. Referring to Figure 9b, the deviation of the measured flow from the reference flow velocity shows a progressive decrease as the flow velocity increases. The different levels of deviation from the reference velocity shown in Figure 9 are very likely attributable to various disturbances in the flow velocity profile. The RMS deviations of the measured flow velocities from the reference velocities can therefore be calculated, and are shown in Table 1. The RMS deviations range from 3.65% to 8.87%, and the average RMS deviation of the eight ultrasonic paths is 6.90%.

**Figure 10:**
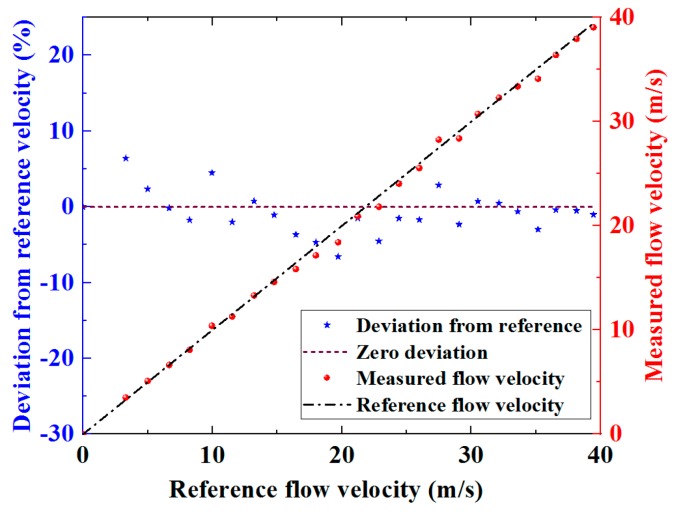
Measured flow velocity as a function of the reference velocity, with associated deviation data, extracted from the spatial averaging method for eight individual ultrasonic paths.

A determination of average flow velocity accounting for the measurements from the eight individual ultrasonic paths as a whole can be obtained using Equation (7). The flow velocity determined by this spatial averaging method and the deviation of this velocity from the reference are both shown in Figure 10.

The results in Figure 10 demonstrate that the spatial averaging method for the measurement of flow velocity based on the flexural ultrasonic array technology significantly reduces the deviation of the measured velocity from the reference. The RMS deviation is approximately 2.94%, which is lower than the deviation of flow velocity measured by any single ultrasonic path, the results for which are shown in Table 1. It has been demonstrated through the results presented in this study that in the acquisition of flow measurement data through individual ultrasonic paths between the single transducer and the array elements, spatial averaging can substantially improve the robustness of the measurements to the fluctuations of fluid flow. It should be noted that the experiments involved in this research do not represent a standard calibration process, which itself can typically be time-consuming and costly, requiring precise configuration and testing under a series of operating conditions. It can be anticipated that, with a rigorous calibration process, the accuracy of the two-dimensional flexural ultrasonic array flow meter will be significantly enhanced further.

A spatial averaging method making full advantage of flow measurement data, extracted utilizing two-dimensional flexural ultrasonic array technology from eight ultrasonic paths in two chordal flow planes, symmetric and adjacent to the diametral plane, was demonstrated. Conventional single-path or multi-path ultrasonic flow meters typically assign only one single or two symmetric ultrasonic paths to each flow plane of interest. The experiments demonstrated in this study show that all of the results, measured respectively via different ultrasonic paths, either located in the same plane or in two symmetric planes, exhibit different levels of discrepancies and errors, which can undermine the measurement accuracy of single-path or multi-path ultrasonic flow meters. By combining the spatial averaging method with flexural ultrasonic array technology, the information of flow velocity in each plane can be expediently extracted through more than one ultrasonic path, ensuring reduced uncertainty in the velocity measurement for the flow plane of interest.

The parameters of this flow measurement configuration, such as the dimensions of the array transducer, the quantity and the positioning of array elements, and the distance between the array transducer and the single transducer, can all be optimized for different flow measurement applications in different diameters of pipe, based on numerical simulation analysis and the orthogonal test method [34]. As the proposed ultrasonic path configuration is formed by an array transducer and a single transducer, the spatial averaging effect varies at different positions on the ultrasonic paths—the closer to the array transducer, the better the spatial averaging effect will be. This configuration could be further improved by replacing the single ultrasonic transducer with another 2 × 4 flexural ultrasonic array transducer, so that invariant three-dimensional spatial averaging can be achieved through all eight ultrasonic paths in parallel. In theory, 64 ultrasonic paths could be formed by two 8-element array transducers, thereby substantially increasing the performance capacity of the flow measurement system and providing the opportunity to implement more sophisticated algorithms. Due to the novel and challenging nature of this research, using one array transducer, operating with a single transducer to prove its feasibility, is the first step necessary to establish the technology. Investigation of configurations consisting of two array transducers will be studied in future research. In practical applications, a compromise between measurement accuracy and the complexities of both the flow measurement configuration and the computational algorithms is necessary. These factors directly affect the design of an ultrasonic flow meter. The array control and signal acquisition FPGA-based system, which has been specifically designed for operating and controlling the flexural ultrasonic array transducer, enables a balance between measurement accuracy and expediency [30,31]. Fundamentally, the flexural ultrasonic array transducer is a relatively inexpensive but robust and reliable solution, due to the inherent simplicity of its structure, and it can therefore be particularly advantageous for a wide range of industrial flow measurement applications.

## 4. Conclusions

This study has demonstrated an innovative solution to enhance the accuracy of the measurement of flow velocity through the implementation of a spatial averaging method, based on the information extracted by two-dimensional flexural array technology. A two-dimensional flexural ultrasonic array transducer was used to conduct flow measurements in a commercial flow rig, utilizing a calibrated mechanical flow meter as a reference. The array transducer comprised eight individual elements positioned in a 2 × 4 configuration, arranged symmetrically around the diametral plane of a custom meter body. This array transducer was operated together with a single ultrasonic transducer, forming eight ultrasonic paths in two chordal planes, symmetric and adjacent to the diametral plane, and measured flow velocities, independently utilizing the classical ultrasonic transit-time flow measurement method. The flow rate was adjusted from 0 to 2500 m^3^/h in steps of 100 m^3^/h, and the average flow velocity over the cross-section of the meter body was thus measured through the eight ultrasonic paths. Fluctuations in measurement were detected around the reference velocity for all eight ultrasonic paths, with RMS deviations ranging from 3.65% to 8.87%, with an average RMS deviation of approximately 6.90%. The spatial averaging method was then implemented on the eight ultrasonic paths as a whole, where the RMS deviation was determined to have reduced to 2.94%. The spatial averaging method was shown to substantially reduce the influence of the fluctuations in flow velocity. Furthermore, the symmetry of the two-dimensional array transducer renders the measurements less susceptible to inaccuracy due to the circumferential flow. Unlike conventional single ultrasonic transducers, which typically form only one single ultrasonic path in a diametral or a chordal plane of a pipe, two-dimensional flexural ultrasonic array technology enables flow velocity to be more accurately measured through multiple adjacent ultrasonic paths in the same plane or two symmetric planes, lowering the uncertainty of measurement. This research is beneficial for both single-path and multi-path ultrasonic flow meters, which typically assign only one ultrasonic or two symmetric paths to each plane of interest. Optimization of the structure and the parameters of the two-dimensional flexural ultrasonic array transducer is possible to further improve the measurement accuracy, provided that the spatial averaging method is properly implemented.

The project website with full details of the research programme with experimental data can be accessed via the following link: https://warwick.ac.uk/fac/sci/physics/research/ultra/research/hiffut/. 

## Figures and Tables

**Table 1:** Root-mean-square (RMS) deviations of the measured flow velocities from the reference velocities through eight paths of ultrasound propagation.

Path	1	2	3	4	5	6	7	8
RMS deviation	7.58%	8.63%	8.87%	5.80%	8.38%	5.96%	6.30%	3.65%
Average RMS deviation	6.90%							
